# Association of Postoperative Hyperamylasemia With Clinically Relevant Postoperative Pancreatic Fistula in Pancreatoduodenectomy

**DOI:** 10.7759/cureus.53257

**Published:** 2024-01-30

**Authors:** Yanagandula Shasheendra, Zeeshan Ahmed, Mahesh G Shetty, Nadendla Hazarathaiah, Pradeep Rebala, Guduru V Rao

**Affiliations:** 1 Surgical Gastroenterology, Asian Institute of Gastroenterology, Hyderabad, IND

**Keywords:** complications, pancreatitis, pancreatoduodenectomy, postoperative pancreatic fistula, postoperative hyperamylasemia

## Abstract

Background

In this study, we aimed to determine the association between postoperative hyperamylasemia (POH) and clinically relevant postoperative pancreatic fistula (CR-POPF) after pancreatoduodenectomy (PD).

Methodology

A prospective observational study of 140 consecutive PDs between March 2020 and March 2022 was conducted. POH was defined as an elevation in serum pancreatic amylase levels above the institutional upper limit of normal on postoperative day (POD) 1 (>100 U/L). CR-POPF was defined as the International Study Group of Pancreatic Surgery Grade B or C POPF. The primary outcome was the rate of CR-POPF in the study population. The trial was prospectively registered with Clinicaltrials.gov (NCT04514198).

Results

In our study, 93 (66.42%) patients had POH (serum amylase >100 U/L). CR-POPF developed in 48 (34.28%) patients: 40 type B and 8 type C. CR-POPF rate was 43.01% (40/93) in patients with POH compared to 17.02% (8/47) in patients without POH (p = 0.0022). Patients with POH had a mean serum amylase of 422.7 ± 358.21 U/L on POD1 compared to 47.2 ± 20.19 U/L in those without POH (p < 0.001). Serum amylase >100 U/L on POD1 was strongly associated with developing CR-POPF (odds ratio = 3.71; 95% confidence interval = 1.31-10.37) on logistic regression, with a sensitivity and specificity of 83.3% and 42.4%, respectively. Blood loss >350 mL, pancreatic duct size <3 mm, and elevated POD1 serum amylase >100 U/L were predictive of CR-POPF on multivariate analysis (p < 0.001).

Conclusions

An elevated serum amylase on POD1 may help identify patients at risk for developing POPF following PD.

## Introduction

Whipple’s pancreatoduodenectomy (PD)-related mortality has significantly decreased (1%-5%) in high-volume centers, but postoperative morbidity is still as high as 20% to 60% [1]. A leak from the pancreatojejunostomy site leading to postoperative pancreatic fistula (POPF) is one of the main culprits for increased morbidity. The risk factors for POPF include small pancreatic duct, soft pancreatic texture, major intraoperative blood loss, ischemia, surgical technique, etc. [2,3].

Background post-pancreatectomy acute pancreatitis (PPAP) has been propounded as a major pathophysiology of POPF in a significant number of patients [4,5]. Initially, there was no uniform definition of PPAP. The first attempt at a standard definition of PPAP was made by Connor et al. in 2016 who defined PPAP as the elevation of serum amylase levels (hyperamylasemia) above the institutional upper limit of normal on postoperative days (POD) 0 or 1 [6]. Several retrospective studies have found postoperative hyperamylasemia of POD0 or 1 to be significantly associated with a higher rate of POPF [7-12].

A universal definition of PPAP was provided by the International Study Group of Pancreatic Surgery (ISGPS) in 2022 wherein they defined PPAP as an acute inflammatory condition of the pancreatic remnant beginning within the first three postoperative days after a partial pancreatic resection [13]. However, a recently published retrospective study from Karolinska Institute in Sweden of 1,078 patients who underwent Whipple’s PD found that the ISGPS criteria may underdiagnose post-pancreatectomy acute pancreatitis because it ignores transiently elevated serum amylase on POD1 and 2 and only considers sustained hyperamylasemia for more than 48 hours postoperatively as significant. Additionally, it requires imaging which is not routinely done in all patients postoperatively and might miss early changes of acute pancreatitis [14].

Therefore, the association of post-pancreatectomy hyperamylasemia with POPF needs further evaluation. To our knowledge, this is the first prospective study to evaluate this association. The study aimed to determine the association between postoperative hyperamylasemia and pancreatic fistula. The objective of the study was to determine the incidence of clinically relevant postoperative pancreatic fistula (CR-POPF), i.e., ISGPS Grade B or C POPF, after pancreaticoduodenectomy, the role of serum amylase levels on day one to predict CR-POPF, and risk factors for CR-POPF.

## Materials and methods

Study design

A prospective observational study was conducted at a tertiary referral center in India. Patients undergoing Whipple’s PD who met the eligibility criteria were enrolled from March 2020 to March 2022. The study was prospectively registered with Clinicaltrials.gov (NCT04514198) and approved by the Institute Ethics Committee. This study was HIPAA-compliant, adhered to the tenets of the Declaration of Helsinki, and written informed consent was obtained from each study participant before enrolment.

All patients (>18 years) undergoing elective Whipple’s PD were included in the study. Patients with cholangitis or bilirubin >15 mg/dL, patients not giving consent for participation, patients with acute inflammatory states (cholangitis, sepsis, trauma, acute on chronic pancreatitis), and on-table inoperable patients were excluded.

Study procedure

Baseline demographic, clinical, pathological, preoperative, intraoperative, and postoperative data were recorded using separate study proforma. All patients were operated on and treated according to the standard Whipple’s PD protocol at the Asian Institute of Gastroenterology, Hyderabad, Telangana, i.e., Heidelberg technique of pancreatojejunostomy, modified Blumgart’s technique for hepaticojejunostomy, and retrocolic-infracolic gastrojejunostomy. All operating surgeons were experienced pancreatic surgeons and followed the standardized operative protocol.

The amylase levels measured in the study were total serum amylase. Postoperative complications were prospectively recorded according to the Clavien-Dindo classification and ISGPS classification. CR-POPF was defined as ISGPS Grade B or C POPF. Clavien-Dindo Grades III-V were considered clinically significant.

Serum amylase was systematically measured on POD1 and 3 according to our institutional policy. Postoperative hyperamylasemia (POH) was defined as an elevation in serum pancreatic amylase above the institutional upper limit of normal on POD1. At our institution, the upper limit of normal for serum pancreatic amylase was 100 U/L.

Primary endpoints were the incidence of POH and CR-POPF. The secondary endpoints were to identify the risk factors of POPF and evaluate the association between POH and CR-POPF.

Statistical analysis

The data for the present study were collected using a separate study proforma and entered into MS Excel (Microsoft Corp., Redmond, WA, USA). The continuous variables were expressed as mean and standard deviation (SD), odds ratio (OR), median, and interquartile range (IQR). The categorical variables were expressed as % of frequency distribution. Student’s t-test, Student’s paired t-test, median test, and chi-square test were used appropriately. Univariate logistic regression for risk factors of developing POPF and multiple logistic regression models were used. The significant risk factors were included in multiple logistic regression. The method of entry of factors was stepwise and two-sided p-value <0.05 was considered statistically significant. The SPSS version 21 (IBM Corp., Armonk, NY, USA)) and MedCalc were used for the analysis.

## Results

Patient characteristics

Our study group had 140 participants (105 males, 35 females) who underwent elective Whipple’s PD. The mean age was 53.5 ± 11.8 years (range = 11-70). The mean basal metabolic index (BMI) was 23.6 ± 3.04 kg/m^2^ (range = 17-33). The mean preoperative bilirubin was 2.86 ± 3.62 mg/dL (range = 0.3-18.0). In 48% of patients, the diagnosis was periampullary adenocarcinoma, 23% of patients had carcinoma head of the pancreas, 7% had distal cholangiocarcinoma, 8% had neuroendocrine tumors, 14% had other benign causes such as serous cystic neoplasms, groove pancreatitis, and inflammatory mass. Preoperative biliary drainage was done in 56% of the patients. The indications for preoperative biliary drainage were serum bilirubin >15 mg/dL, cholangitis, borderline resectable pancreatic cancer, and severe malnutrition. Mean intraoperative blood loss was 361 ± 154.5 mL (range = 30-1,000). The texture of the pancreas was soft in 61% of the patients, 45% had a posteriorly placed duct, and 26% had <3 mm pancreatic duct (small duct). The mean pancreatic duct size was 4.58 ± 2.61 mm (range = 1-22).

Postoperative complications

The mean postoperative intensive care unit (ICU) stay was 4.8 ± 2.49 days (range = 3-16), and the mean postoperative stay was 12.9 ± 4.77 days (range = 7-32). We had 58% morbidity, out of which 21% were major complications (Clavien-Dindo Grade ≥3). Our mortality rate was 2.85%. CR-POPF rate was 34.3% (Table 1).

**Table 1:** Postoperative complications in the study population. CR-POPF = clinically relevant postoperative pancreatic fistula; DGE = delayed gastric emptying; PPH = post-pancreatectomy hemorrhage; SSI = surgical site infections; CD = Calvien-Dindo

Complication	n	%
CR-POPF	48 (B = 40, C = 8)	34.3
DGE	41	29.3
PPH	17	12.1
SSI	14	10
Sepsis	28	18.6
Minor (CD Grade <3)	64	45.7
Major (CD Grade ≥3)	30	21

Postoperative hyperamylasemia

POD1 hyperamylasemia (POH) was found in 93/140 (66.43%) patients with a mean serum amylase of 422.7 ± 358.21, while the mean serum amylase in the 47/140 (33.57%) patients with normal POD1 amylase was 47.2 ± 20.19 (p = 0.0001). POD3 hyperamylasemia was found in 37/124 (29.83%) patients with a mean serum amylase of 202.2 ± 103.67, while the mean serum amylase was 44.8 ± 22.67 (p = 0.0001) in patients with normal serum amylase (Table 2).

**Table 2:** Serum amylase levels on POD1 and 3. POD = postoperative day; SD = standard deviation

Serum amylase levels	n (%)	Mean ± SD
POD1 serum amylase (U/L)	140 (100)	295.8 ± 341.3
POD1 serum amylase <100 U/L	47 (33.57)	47.2 ± 20.19
POD1 serum amylase >100 U/L	93 (66.43)	422.7 ± 358.21
POD3 serum amylase (U/L)	124	91.8 ± 93.5
POD3 serum amylase <100 U/L	87 (70.16)	44.8 ± 22.67
POD3 serum amylase >100 U/L	37 (29.83)	202.2 ± 103.67

Analysis of risk factors for POPF

Univariate Analysis

Age, BMI, preoperative bilirubin levels, blood loss during surgery, serum amylase on POD1 and 3, pancreatic duct size, pancreatic texture, preoperative biliary drainage, surgery duration, and diagnosis were analyzed. A soft pancreatic texture, a main pancreatic duct diameter <3 mm, blood loss of more than 350 mL, non-carcinoma head of the pancreas diagnosis, and the occurrence of POAP were independent predictors of POPF (p < 0.05) (Table 3).

**Table 3:** Univariate analysis of risk factors for postoperative pancreatic fistula. BMI = basal metabolic index; CI = confidence interval; PD = pancreatic duct; CHOP = carcinoma head of the pancreas

Risk factor		Odds ratio	P-value	95% CI
Age (year)	<55	1.29	0.47	0.6-2.6
BMI (kg/m^2^)	>25	1.39	0.411	0.63-3.06
Preoperative bilirubin (mg/dL)	<1	0.84	0.632	0.41-1.71
Preoperative biliary drainage	No	1.42	0.326	0.7-2.86
Diagnosis	CHOP	0.21	0.005	0.06-0.63
Duration of surgery (hour)	>8	1.25	0.577	0.56-2.79
Blood loss (mL)	>350	3.77	0.001	1.78-7.99
PD size (mm)	<3	3.22	0.003	1.46-7.07
Pancreatic texture	Soft	3.48	0.002	1.55-7.81
Serum amylase day 1 (U/L)	>100	4.39	0.001	1.78-10.84
Serum amylase day 3 (U/L)	>100	2.61	0.017	1.18-5.79

Multivariate Analysis

Stepwise multivariate analysis of risk factors showed blood loss >350 mL, main pancreatic duct size (<3 mm), and POH predictive of CR-POPF (p <0.001) (Table 4).

**Table 4:** Multivariate analysis of risk factors for postoperative pancreatic fistula. POD = postoperative day; SD = standard deviation; PD = pancreatic duct

Risk factor	Coefficient	Standard error	Odds Ratio	P-value	95% CI
Blood loss >350 mL	1.229	0.424	3.42	0.003	1.48-7.86
PD size <3 mm	0.965	0.463	2.62	0.037	1.05-6.51
Serum amylase >100 U/L (POD1)	1.308	0.526	3.71	0.012	1.31-10.37

Association between POH and POPF

Of the 93 patients who developed POH, CR-POPF developed in 40 (43.01%) patients. Ultrasound-guided single-time aspiration and percutaneous drainage for intra-abdominal collections were done in 10 and 19 nineteen patients, respectively. Angioembolization for PPH was done in six patients. Reoperation for Grade C POPF was done in two patients, whereas three patients were managed in the ICU for septic shock with multiorgan failure, of whom two patients expired. Of the 47 patients who did not develop POH, eight (17.02%) had CR-POPF. There was a significantly higher rate of CR-POPF in patients who had POH on POD1 (p = 0.0022). The sensitivity and specificity of serum amylase levels >100 U/L on POD1 to predict CR-POPF were 83.3%, and 42.4%, respectively. Positive predictive value, negative predictive value, and accuracy were 43%, 82.9%, and 56.43%, respectively.

## Discussion

Our POH and CR-POPF rates were 66.4% and 34.3%, respectively, which were comparable to POH and POPF rates of 55.8% and 22.3%, respectively, in a study by Bannone et al. [10]. In a retrospective study of 190 patients who underwent PD, 53% developed POH, and 19% had Grade B or C complications [11]. Loos et al. showed a POH rate of 52% of patients after pancreatoduodenectomy [12]. Chen et al. showed a POH rate of 53% and a CR-POPF rate of 19% in a retrospective study of 1,465 PDs [15].

This study suggests that POH is closely associated with POPF which is similar to several previously published retrospective studies. Radulova et al. showed a POH1 rate of 52.3% in 437 patients, with soft pancreatic texture and POH on POD1 being independent predictors of POPF. The CR-POPF, major complications (Clavien-Dindo classification > 2), and completion pancreatectomy rates were significantly higher in the POH group [8]. Kuhlbrey et al. showed in a retrospective study of 739 consecutive PDs that POD1 systemic hyperamylasemia was a good predictor for CR-POPFs [16].

Our study showed that the sensitivity and specificity of serum amylase levels >100 U/L on POD1 to predict CR-POPF were 83.3%, and 42.4%, respectively. Cloyd et al. showed in a retrospective study of 146 consecutive PDs that POD1 serum amylase levels >140 U/L were strongly associated with developing a POPF with sensitivity and specificity of 81.5% and 55.5%, respectively [7]. The predictive factors for POPF were blood loss >350 mL, main pancreatic duct size <3 mm, and POH in our study. Palanivelu et al. showed that POD0 serum amylase levels of ≥130 IU/L and soft pancreatic parenchyma were independent risk factors for CR-POPF in a retrospective study of 240 PDs [5].

ISGPS published a consensus definition and grading for PPAP in 2022 [13]. After the publication of this criteria, several studies have been published to validate this definition. Chen et al. conducted a retrospective study of 716 patients, in which PPAP occurred in 152 (21.2%) patients with a significantly higher rate of POPF and major complications in the PPAP group [17]. Wu et al. showed the incidence of PPAP was 52.4% (150 patients) in a retrospective study of 298 patients with significantly increased severe complications and mortality in PPAP associated with pancreatic fistula [18].

On the other hand, the clinical relevance of the new ISGPS PPAP definition has been questioned. Holmberg et al. conducted a retrospective single-center observational study of 1,078 patients who underwent Whipple’s PD. Sustained (>48 hours postoperatively) hyperamylasemia and transient hyperamylasemia (on either POD1 or 2) were found in 284 (26%) and 183 (17%) patients, respectively. The rate of major complications (Clavien-Dindo Grade >2) in the sustained and transient hyperamylasemia group was 43% (n = 123) and 32% (n = 58), respectively. Interestingly, only 18 (6.3%) patients in the sustained hyperamylasemia group showed radiological evidence of acute pancreatitis. The authors concluded that the current ISGPS criteria may underdiagnose PPAP by ignoring transient hyperamylasemia and excluding patients with no radiological evidence of acute pancreatitis [14].

The strength of this study is that it was a prospective study of a population of Indian patients undergoing PD at a tertiary referral center. On the other hand, a relatively small sample size of 140 patients and a lack of data on radiological imaging according to the ISGPS criteria are the main limitations of this study. Our study started before the publication of the ISGPS criteria and we did not include routine postoperative radiological imaging as part of our protocol, although they were done as clinically indicated in patients.

## Conclusions

An elevated serum amylase on POD1 may be a useful predictor of clinically relevant pancreatic fistula in addition to other prognostic factors such as intraoperative blood loss (>350 mL) and small (<3 mm) pancreatic duct. Further prospective studies are needed to validate our findings.
